# Oral health-related quality of life among people living with HIV and HIV-negative adults in Kigali, Rwanda: a comparative cross-sectional study

**DOI:** 10.1186/s12903-023-03828-9

**Published:** 2024-01-25

**Authors:** Julienne Murererehe, Yolanda Malele-Kolisa, François Niragire, Veerasamy Yengopal

**Affiliations:** 1 Department of Preventive and Community Dentistry, University of Rwanda, Kigali, Rwanda; 2 Department of Community Dentistry, University of Witwatersrand, Johannesburg, South Africa; 3 Department of Applied statistics, University of Rwanda, Kigali, Rwanda; 4 Department of Community Dentistry, University of Western Cape, Cape town, South Africa

**Keywords:** HIV, Oral health-related quality of life, Oral health impact profile, Adult, Rwanda

## Abstract

**Background:**

Assessing health-related quality of life has become integral to people living with HIV (PLHIV) follow-up. However, there is a lack of data regarding the impact of oral health on quality of life, known as Oral health-related quality of life (OHRQoL) among PLHIV compared to HIV-negative individuals in Rwanda.

**Aim:**

The study aimed to assess OHRQoL among PLHIV compared to HIV-negative counterparts in Kigali, Rwanda.

**Methods:**

The Oral Health Impact Profile short version (OHIP-14) questionnaire was interviewer-administered to 200 PLHIV and 200 HIV-negative adults (≥ 18 years old) at an HIV clinic of Kigali Teaching Hospital (CHUK). Socio-demographic characteristics, including age, sex, occupation, and socioeconomic status (SES) of participants, were collected using a survey questionnaire. A 4-point Likert scale was used to assess the frequency of oral impacts for all 14 items within 7 domains of the OHIP tool. The descriptive statistics were used to see frequencies and percentages of OHRQoL among PLHIV and HIV-negative persons, respectively. The Chi-square test was used to determine the association of OHRQoL with caries (DMFT) and periodontal disease (CPITN) among PLHIV compared to HIV-negative individuals.

**Results:**

The results revealed a higher prevalence of PLHIV with poor OHRQoL than HIV-negative individuals in 5 domains and almost all items of OHIP-14 except for the OHIP 3 (found it uncomfortable to eat any foods because of problems with teeth or mouth) and OHIP-14 (being totally unable to function because of problems with teeth or mouth). The findings showed statistically significant results (*p* ≤ 0.05) for the OHIP1 item “trouble pronouncing any word,” with a prevalence of 2.5% (*n* = 11) and 2.25% (*n* = 9) in PLHIV and HIV-negative, respectively. Also, PLHIV had a significantly higher prevalence, 2.75% (*n* = 11) for the OHIP 13 item “life not satisfying due to teeth and mouth problems compared to HIV-negative individuals 2% (8) *p* ≤ 0.05. Moreover, dental caries was significantly associated with poor OHRQoL among PLHIV and HIV-negative adults and for all 14 items of the OHIP tool. Periodontal disease was not significantly associated with OHRQoL among PLHIV and HIV-negative adults.

**Conclusion:**

This study revealed poor OHRQoL among PLHIV compared to HIV-negative adults. There is a need for further longitudinal studies to investigate the OHRQoL in Rwanda, especially among PLHIV. It is essential to include oral health care as one of the components of the medical health care programs for PLHIV in Rwanda.

## Background

Human Immune Deficiency Virus (HIV) infections remain a global health crisis [1]. About 680,000 people died from AIDS-related causes in 2020 [2]. Although overshadowed by the more recent COVID-19 pandemic, almost 4000 new HIV infections are reported worldwide [3]. Among those, 60% of daily new HIV infections occur in Sub-Saharan Africa [3]. Recently, the 90–90-90 targets (by 2020, (1) 90% of all people living with HIV will know their HIV status; (2) 90% of all people diagnosed with HIV infection will receive sustained antiretroviral therapy; (3) 90% of all people receiving antiretroviral therapy will have viral suppression) have contributed to an increased number of HIV-infected individuals with suppressed viral load [3] and having an increased life expectancy [2]. As People Living with HIV (PLHIV) live longer, controlling other chronic diseases, such as oral diseases, that hinder their quality of life is paramount.

Good oral health is crucial to a better quality of life. The World Health Organization (WHO) distinguishes the Oral Health Related Quality of Life (OHRQoL) concept as essential to general and oral health [4]. Also, WHO recognizes oral health as a significant part of the Global Oral Health Program [4]. For years, the impact of oral diseases on quality of life was ignored, and more effort was put into evaluating oral diseases clinically [5]. However, oral conditions, mostly caries and periodontal disease, are reported as a highly prevalent group of pathologies globally [6], and have been shown to affect the ability to eat, speak, smile, and psychological status [4].

The literature defines OHRQoL as “a multidimensional construct that reflects people’s comfort when eating, sleeping, and engaging in social interaction, their self-esteem, and their satisfaction concerning their oral health” [7, 8]. The definition of OHRQoL is mostly based on its constructs: functional factors (mastication, speaking); psychological factors (appearance, self-esteem); social factors (intimacy, communication) and experience of pain (acute or chronic) or discomfort [8].

Caries and periodontal diseases negatively affect patients’ OHRQoL and well-being. Oral conditions affect OHRQoL through interference with daily activities such as chewing, swallowing, speaking, sleeping, choosing what to eat, schooling social interaction, and more [4]. Poor oral health negatively affects the quality of life generally [5], and especially among people with other chronic diseases such HIV-infection [9]. For example, pain and discomfort caused by dental caries and periodontal diseases an interfere with nutritional intake among PLHIV and, therefore, can affect their immunity and general well-being [10].

Assessing the health-related quality of life became integral to PLHIV follow-up [11]. However, there is a lack of data regarding the impact of oral health on quality of life among PLHIV compared to HIV-negative individuals in Rwanda. Elsewhere, studies have reported poorer OHRQoL especially among high risk group such as PLHIV [12].

Thus, this study aimed to assess Oral health related quality of life among PLHIV compared to HIV-negative adults in Kigali, Rwanda. The results from this study will provide much-needed data for stakeholders to contribute to improvement of oral health generally and for PLHIV in Rwanda. It may also guide other researchers in Rwanda to include an OHRQoL component in their future oral health research.

## Methods

### Study design and participants

This study used a comparative cross-sectional design comprising People living with HIV (PLHIV) and HIV-negative adults aged ≥18. Study participants were recruited from an HIV Clinic of Kigali Teaching Hospital (CHUK) from August 2020 to December 2021.

### Study setting

The study was conducted at an HIV clinic in the University Teaching Hospital of Kigali (CHUK). CHUK is the largest tertiary national referral hospital located in Kigali City, Rwanda. It is also one of the biggest referral hospitals in the country. According to the records, around 3150 PLHIV were seen monthly for routine treatment and management of their condition. Also about 106 HIV-negative persons were received for HIV voluntary testing.

### Recruitments process

A consecutive sampling method was used to select study participants. PLHIV were recruited next to the physicians’ rooms after being informed about the ongoing study by the nurse or physician. The PLHIV signed the consent form before data collection process. HIV-negative adults were recruited in the room next to the Counselling and Testing site (VCT). HIV-negative participants were first given results by their physicians or nurses, and they were informed about the ongoing study. Only HIV-negative people who agreed to participate were guided to data collectors to sign the consent before starting data collection.

### Sample size

The sample for this study was delivered from a broader PhD study that sought to assess risk factors for caries and periodontal diseases among PLHIV and HIV-negative adults in Rwanda. The sample size calculation for the comparative cross-sectional study was done using Stata software version 15. Assuming a study power of 80%, considering that the prevalence of common oral diseases is 60% in PLHIV and 45% in HIV-negative individuals, and considering a ratio of 1 [13], the minimum study sample was estimated to be 346. Four hundred patients, 200 PLHIV and 200 HIV-negative adults, were recruited and agreed to participate in the study.

### Data collection tools and procedure

To assess OHRQoL, oral health impact profile short version (OHIP-14) tool [14] was interviewer-administered to study participants in the language they could understand. The OHIP tool uses seven variable dimensions developed from an oral health model by Locker and consists of 49 questions subdivided into seven dimensions [14]. Slade and Spencer later developed the OHIP-14, a short version instrument to assess OHRQoL [14]. The OHIP-14 instrument is the most widely used and comprises seven domains (functional limitation, physical pain, psychological discomfort, physical disability, psychological disability, social disability, and handicap) and each dimension has 2 items [14].

We used 4-point Likert scale responses to assess the frequency of oral impacts during the previous 3 months for all domains. The coding for 4-point Likert was ‘0’ for ‘never,’ ‘1’ for ‘hardly ever,’ ‘2’ for ‘occasionally’, ‘3’ for ‘fairly often,’ and ‘4’ for ‘very often. A 4-point Likert scale was used to assess the frequency of oral impacts during the previous 3 months for all domains.

To facilitate the interpretation of our results, participants’ responses on the OHIP-14 questionnaire for “fairly often” (coded 3) and “very often” (coded 4) were combined to present high impact. “Occasionally” (coded 2) was counted on its own as neutral, while “hardly ever” (coded 1) and “never” (coded 0) were also added together to represent low impact. Higher impact indicates worse OHRQoL. The training of data collector on how to use OHIP-14 tool was provided before data collection.

Socio-demographic characteristics, including age, sex, occupation, and socioeconomic status (SES) of participants, were also collected using a survey questionnaire. In Rwanda context socio-economic status is already known because it is pre-determined by the government criteria and every Rwandese knows the category he or she belongs too. Those categories are defined as follow:

- *Category 1*: Families who do not own a house and can hardly afford basic needs.
- *Category 2* Those who have a dwelling of their own or are able to rent one but rarely get full time jobs.
- *Category 3*: Those who have a job and farmers and go beyond subsistence farming to produce a surplus which can be sold. Also include those with small and medium enterprises who can provide employment to dozens of people.
- *Category 4*: Those who own large-scale business, individuals working with international organizations and industries as well as public servants.

Age was stratified as 18–24; 25–34; 36–45; 46–55 and 56+. For easy of comparison, we based on existing literature to categorize participants’ age [15]. Age was assessed through strata.

We also used two clinical indices: DMFT (Decayed, Missing, and filled) index following WHO guidance [16] and CPITN (Community Periodontal Index of Treatment Need) [17] to determine and compare the association of caries (DMF) and periodontal disease (CPITN) with OHRQoL among PLHIV and HIV-negative adults. Clinical data (DMF and CPITN) information were delivered from the broader PhD project that aimed to assess risk factors for caries and periodontal diseases among PLHIV and HIV-negative persons in Kigali, Rwanda.

DMFT and CPITN were categorized as yes or no. Yes, indicates participants who experienced dental caries /in need of periodontal treatment and no expressed those who did not experience dental caries/no need of periodontal treatment. The explanatory variables were age, sex, education, residence, socio-economic status (SES), caries experience, and periodontal disease (CPITN). The outcome variable was Oral health-related quality of life (OHRQoL).

### Statistical analysis

The descriptive statistics were used determine frequencies and percentages of OHRQoL among study participants. The chi-square test was done to assess and compare the association between OHRQoL and participants’ characteristics, caries (DMFT), and periodontal disease (CPITN). The Cronbach alpha test was performed to test the internal consistency of the OHIP-14 tool in Rwanda, and the alpha coefficient scored 0.914. Stata version 15 was used to analyze data.

## Results

### Demographic characteristics of participants

According to results in Table 1, majority of people living with HIV (PLHIV) showed higher prevalence in older age categories of 46–55 [28,5% (*n* = 57)] and 56+ [24% (*n* = 54)] compared to HIV-negative participants in 46–55 [(11.5% (*n* = 23)] and 56+ [12% (*n* = 21)] age categories. In addition, 32% (*n* = 64) of PLHIV were unemployed, whereas in the HIV-negative group this percentage was 22% (*n* = 44) (*p* = 0.024). Furthermore, the prevalence of PLHIV in socioeconomic status categories 3 and 4 (considered as having enough income) was significantly higher (67.5%) than the prevalence of HIV-negative respondents in the same socioeconomic status (51.5%) (*p* = 0.005).

**Table 1:** Demographic characteristics of participants

Social demographic characteristics	PLHIV n(%)	HIV-negative n(%)	*P* value
**Age**			< 0.001*
18–24	30 (15)	39 (19.5)	
25–35	31 (15.5)	77 (38.5)	
36–45	34 (17.0)	39 (19.5)	
46–55	57 (28.5)	23 (11.5)	
56+	54 (24.0)	21 (12.0)	
**Sex**			
Male	88 (44.0)	89 (44.5)	0.920
Female	112 (56.0	111 (55.5)	
**Occupation**			
Unemployed	64 (32.0)	44 (22.0)	0.024*
Employed	136 (68.0)	156 (78.0)	
**Socio-economic status**			
Category1	18 (9.0)	24 (12.0)	0.005*
Category2	47 (23.5)	73 (36.5)	
Category3 and 4	135 (67.5)	103 (51.5)	

^*^Indicator of statistical significant results with a significance level of 0.05

### Comparison of OHIP frequency in PLHIV and HIV-negative adults

Based on the “high impact” category for all 14 items of OHIP, the results in Table 2 revealed a higher percentage of high impact among PLHIV than HIV-negative people in 5 domains (functional limitation, psychological discomfort, physical disability, psychological disability, social disability) and almost all items of OHIP-14 except for OHIP 3 (found it uncomfortable to eat any foods because of problems with teeth or mouth) and OHIP-14 (being totally unable to function because of problems with teeth or mouth) that were within the domain of physical pain and Handicap, respectively. The findings revealed statistically significant results (*p* ≤ 0.05) for the OHIP1 item “trouble pronouncing any word,” with a prevalence of 2.5% (*n* = 11) and 2.25% (*n* = 9) in PLHIV and HIV-negative persons, respectively. Also, considering the OHIP 13 item “life not satisfying due to teeth and mouth problems”, the results showed a higher prevalence of impact among PLHIV (2.75%) when compared to HIV-negative individuals (2%). The difference was statistically significant *p* ≤ 0.05.

**Table 2:** Comparison of OHIP frequency among study participants according to HIV status

7 domain	Items of OHIP	Categories impact in terms of OHRQoL	HIV-negative n(%)	PLHIV n(%)	*p*-value
Functional limitation	OHIP1: Had trouble pronouncing any words	low impact	195 (48.75)	180 (45)	**0.008**
		neutral impact	3 (0.75)	11 (2.75)	
		high impact	2 (0.5)	9 (2.25)	
	OHIP2: Felt sense of taste has worsened	low impact	173 (43.25)	158 (39.5)	0.091
		neutral impact	16 (4)	20 (5)	
		high impact	11 (2.75)	22 (5.5)	
Physical pain	OHIP3: Had painful aching	low impact	99 (24.75)	91 (22.75)	0.559
		neutral impact	45 (11.25)	54 (13.5)	
		high impact	56 (14)	55 (13.75)	
	OHIP4: Find it uncomfortable to eat any foods	low impact	124 (31)	105 (26.25)	0.06
		neutral impact	41 (10.25)	41 (10.25)	
		high impact	35 (8.75)	54 (13.5)	
Psychological discomfort	OHIP5: Been self-conscious	low impact	143 (35.75)	122 (30.5)	0.082
		neutral impact	25 (6.25)	36 (9)	
		high impact	32 (8)	42 (10.5)	
	OHIP6: Felt tense	low impact	140 (35)	123 (30.75)	0.173
		neutral impact	27 (6.75)	31 (7.75)	
		high impact	33 (8.25)	46 (11.5)	
Physical disability	OHIP7: Felt diet has been unsatisfactory	low impact	138 (34.5)	131 (32.75)	0.619
		neutral impact	34 (8.5)	34 (8.5)	
		high impact	28 (7)	35 (8.75)	
	OHIP8: Had to interrupt meal	low impact	151 (37.75	136 (34)	0.173
		neutral impact	27 (6.75)	30 (7.5)	
		high impact	22 (5.5)	34 (8.5)	
Psychological disability	OHIP9: Found it difficult to relax	low impact	137 (34.25)	133 (33.25)	0.669
		neutral impact	36 (9)	34 (8.5)	
		high impact	27 (6.75)	33 (8.25)	
	OHIP10: Been a bit embarrassed	low impact	161 (40.25)	154 (38.5)	0.469
		neutral impact	19 (4.75)	18 (4.5)	
		high impact	20 (5)	28 (7)	
Socio disability	OHIP11: Been a bit irritable	low impact	168 (42)	164 (41)	0.139
		neutral impact	19 (4.75)	13 (3.25)	
		high impact	13 (3.25)	23 (5.75)	
	OHIP12: Had difficult doing usual jobs	low impact	163 (40.75)	155 (38.75)	0.216
		neutral impact	24 (6)	22 (5.5)	
		high impact	13 (3.25)	23 (5.75)	
Handicap	OHIP13: Felt life not satisfying	low impact	183 (45.75)	162 (40.5)	**0.005**
		neutral impact	9 (2.25)	27 (6.75)	
		high impact	8 (2)	11 (2.75)	
	OHIP14: Being totally unable to function	low impact	181 (45.25)	179 (44.75)	921
		neutral impact	12 (3)	14 (3.5)	
		high impact	7 (1.75)	7 (1.75)	

### Association of Oral Health-Related Quality of life (OHRQoL) with demographic characteristics among PLHIV adults in comparison to HIV-negative persons

Females living with HIV reported significantly higher OHIP-14 impact (25%) when compared to males (9.09%). Although not statistically significant, HIV-negative females (10.81%) reported a higher OHIP impact than HIV-negative males (8.99%) as shown in Table 3. In addition, PLHIV (17.99%) and HIV-negative participants (13.10%) in the older age group (36+ years) reported a slightly higher OHIP-14 impact compared to young aged (18–35 years) PLHIV (18.03%) and HIV-negative adults (7.76%). Moreover, employed PLHIV (21.88%) reported significantly higher OHIP-14 impact compared to unemployed PLHIV (16.18%) (*p* = 0,031) as shown in Table 3.

**Table 3:** Association of OHRQoL with demographic characteristics PLHIV in comparison to HIV-negative individuals

Variables	PLHIV			*P* value	HIV-negative			*P* value
	High impact n(%)	Neutral impact n(%)	Low impact n(%)		High impact n(%)	Neutral impact n(%)	Low impact n(%)	
**AGE**								**0.001**
18–35	11 (18.03)	25 (40.98)	25 (40.98)	0.645	9 (7.76)	41 (35.34)	66 (56.90)	
36+	25 (17.99)	66 (47.48)	48 (34.53)		11 (13.10)	48 (57.14)	25 (29.76)	
**SEX**								
Male	8 (9.09)	42 (47.73)	38 (43.18)	**0.011**	8 (8.99)	35 (39.33)	46 (51.69)	**0.0290**
Female	28 (25.00)	49 (43.75)	35 (31.25)		12 (10.81)	54 (48.65)	45 (40.54)	
**•UBUDEHE (SES)**								
Cat 1&2 (poor)	12 (18.46)	29 (44.62)	24 (36.92)	0.984	9 (9.28)	43 (44.33)	45 (46.39)	0.936
Cat 3& 4 (Rich)	24 (17.78)	62 (45.93)	49 (36.30)		11 (10.68))	46 (44.66)	46 (44.66)	
**OCCUPATION**								
employed	14 (21.88)	35 (54.69)	15 (23.44)	**0.031**	4 (99.09)	20 (45.45)	20 (45.45)	0.971
unemployed	22 (16.18)	56 (41.18)	58 (42.65)		16 (10.26)	69 (44.23)	71 (45.51)	

**• **UBUDEHE: mean socio-economic status in Rwanda and its categories are defined as follow:

*Category 1*: Families who do not own a house and can hardly afford basic needs

*Category 2* Those who have a dwelling of their own or are able to rent one but rarely get full time jobs

*Category 3*: Those who have a job and farmers and go beyond subsistence farming to produce a surplus which can be sold. Also include those with small and medium enterprises who can provide employment to dozens of people

*Category 4*: Those who own large-scale business, individuals working with international organizations and industries as well as public servants

### Comparisons of OHIP-14 among PLHIV and HIV-negative adults according to dental caries (DMFT index)

Having experienced dental caries (DMFT> 0) was significantly associated with poorer OHRQoL in all domains of OHIP-14 among PLHIV and HIV-negative adults (Table 4). None of the OHIP-14 items was significantly associated with CPITN as shown in Table 4.

**Table 4:** Comparisons of OHIP-14 among PLHIV and HIV-negative adults according to DMFT index and CPITN

**OHIP items**	**PLHIV**							**HIV-negative**						
	**+DMFT**							**+DMFT**						
	**Yes** **n(%)**			**No** **n(%)**				**Yes** **n(%)**			**No** **n(%)**			
	**High impact**	**Neutral impact**	**Low impact**	**High impact**	**Neutral impact**	**Low impact**	***P*** **value**	**High impact**	**Neutral impact**	**Low impact**	**High impact**	**Neutral impact**	**Low impact**	***P*** **value**
OHIP1	8 (7.92)	11 (10.89)	82 (81.19)	1 (1.01)	0 (0.00)	98 (98.99)	**0.000**	2 (2.47)	3 (3.70)	76 (93.83)	0 (0.00)	0 (0.00)	119 (100.00)	**0.023**
OHIP2	17 (16.83)	15 (14.85)	69 (68.32)	89 (89.90)	5 (5.05)	5 (5.05)	**0.001**	7 (8.64)	11 (13.58)	63 (77.78)	4 (3.36)	5 (4.20)	110 (92.44)	**0.011**
OHIP3	43 (42.57)	40 (39.60)	18 (17.82)	12 (12.12)	14 (14.14)	73 (73.74)	**0.000**	34 (41.98)	31 (38.27)	16 (19.75)	22 (18.49)	14 (11.76)	83 (69.75)	**0.000**
OHIP4	42 (41.58)	30 (29.70)	29 (28.71)	12 (12.12)	11 (11.11)	76 (76.77)	**0.000**	25 (30.86)	28 (34.57)	28 (34.57)	10 (8.40)	13 (10.92)	96 (80.67)	**0.000**
OHI 5	32 (31.68)	26 (25.74)	43 (42.57)	10 (10.10)	10 (10.10)	79 (79.80)	**0.000**	21 (25.93)	15 (18.52)	45 (55.56)	11 (9.24)	10 (8.40)	98 (82.35)	**0.000**
OHIP 6	35 (34.65)	25 (24.75)	41 (40.59)	11 (11.11)	6 (6.06)	82 (82.83)	**0.000**	23 (28.40)	16 (19.75)	42 (51.85)	10 (8.40)	11 (9.24)	98 (82.35)	**0.000**
OHIP 7	29 (28.71)	26 (25.74)	46 (45.54)	6 (6.06)	8 (8.08)	85 (85.86)	**0.000**	19 (23.46)	25 (30.86)	37 (45.68)	9 (7.56)	9 (7.56)	101 (84.87)	**0.000**
OHIP 8	27 (26.73)	26 (25.74)	48 (47.52)	7 (7.07)	4 (4.04)	88 (88.89)	**0.000**	15 (18.52)	22 (27.16)	44 (54.32)	7 (5.88)	5 (4.20)	107 (89.92)	**0.000**
OHIP 9	29 (28.71)	28 (27.72)	44 (43.56)	4 (4.04)	6 (6.06)	89 (89.90)	**0.000**	19 (23.46)	26 (32.10)	36 (44.44)	8 (6.72)	10 (8.40)	101 (84.87)	**0.000**
OHIP 10	20 (19.80)	12 (11.88)	69 (68.32)	8 (8.08)	6 (6.06)	85 (85.86)	**0.012**	14 (17.28)	16 (19.75)	51 (62.96)	6 (5.04)	3 (2.52)	110 (92.44)	**0.000**
OHIP 11	19 (18.81)	10 (9.90)	72 (71.29)	4 (4.04)	3 (3.03)	92 (92.93)	**0.000**	8 (9.88)	17 (20.99)	56 (69.14)	5 (4.20)	2 (1.68)	112 (94.12)	**0.000**
OHIP 12	21 (20.79)	18 (17.82)	62 (61.39)	2 (2.020	4 (4.04)	93 (93.94)	**0.000**	8 (9.88)	19 (23.46)	54 (66.67)	5 (4.20)	5 (4.20)	109 (91.60)	**0.000**
OHIP 13	8 (7.92)	18,917.82)	75 (74.26)	3 (3.03)	9 (9.09)	87 (87.88)	**0.046**	6 (7.41)	6 (7.41)	69 (85.19)	291.68)	3 (2.52)	114 (95.80)	**0.029**
OHIP 14	6 (5.94)	10 (9.90)	85 (84.16)	1 (1.01)	4 (4.04)	94 (94.95)	**0.037**	5 (6.17)	12 (14.81)	64 (79.01)	2 (1.68)	0 (0.00)	117 (98.32)	**0.000**
	**CPITN**						***P*** **value**	**CPITN**						***P*** **value**
	**Yes** **n(%)**			**No** **n(%)**				**Yes** **n(%)**			**No** **n(%)**			
OHIP1	9 (5.20)	10 (5.78)	154 (89,02)	0 (0.00)	1 (3.70)	26 (96.30)	0.422	2 (1.06)	3 (1.59)	184 (97.35)	0 (0.00)	0 (0.00)	9 (100.00)	0.885
OHI2	20 (11.56)	16 (9.25)	137 (79.19)	2 (7.41)	4 (14.81)	21 (77.78)	0.578	9 (4.76)	16 (8.47)	164 (86.77)	1 (11.11)	0 (0.00)	8 (88.89)	0.484
OHIP3	48 (27.75)	46 (26.59)	79 (45.66)	7 (25.93)	8 (29.63)	12 (44.44)	0.944	50 (26.46)	44 (23.28)	95 (50.26)	4 (44.44)	1 (11.11)	4 (44.44)	0.441
OHIP4	47 (27.17)	34 (19.65)	92 (53.18)	7 (25.73)	7 (25.73)	13 (48.15)	0.750	32 (16.93)	40 (21.16)	117 (61.90)	3 (33.33)	1 (11.11)	5 (55.56)	0.410
OHIP5	36 (20.81)	31 (17.92)	106 (61.27)	6 (22.22)	5 (18.52)	16 (59.26)	0.979	30 (15.87)	23 (12.17)	136 (71.96)	2 (212.22)	2 (22.22)	5 (55.56)	0.542
OHIP6	39 (22.54)	27 (15.61)	16 (59.26)	7 (25.93)	4 (14.81)	16 (59.26)	0.927	30 (15.87)	25 (13.23)	134 (70.90)	3 (33.33)	2 (22.22)	4 (44.44)	0.229
OHIP7	29 (16.76)	29 (16.76)	115 (66.47)	6 (22.22)	5 (18.52)	16 (59.26)	0.731	25 (13.23)	32 (16.93)	132 (69.84)	3 (33.33)	2 (22.22)	4 (44.44)	0.182
OHIP8	30 (17.34)	23 (13.29)	120 (69.36)	4 (14.81)	7 (25.93)	16 (59.26)	0.232	20 (10.58)	25 (13.23)	114 (76.19)	2 (22.22)	2 (22.22)	5 (55.56)	0.360
OHIP9	28 (16.18)	30 (17.34)	115 (66.47)	5 (18.52)	4 (14.81)	18 (66.67)	0.921	24 (12.70)	35 (18.52)	130 (68.78)	1 (11.11)	3 (33.33)	5 (55.56)	0.206
OHIP10	25 (14.45)	15 (8.67)	133 (76.88)	3 (11.11)	3 (11.11)	21 (77.78)	0.842	20 (10.58)	17 (8.99)	152 (80.42)	0 (0.00)	2 (22.22)	7 (77.78)	0.283
OHIP11	19 (10.98)	11 (6.36)	143 (82.66)	4 (14.81)	2 (7.41)	21 (77.78)	0.816	12 (6.35)	18 (9.52)	159 (84.13)	1 (11.11)	1 (11.11)	7 (77.78)	0.835
OHIP12	19 (10.98)	17 (9.83)	137 (79.19)	4 (14.81)	5 (18.52)	18 (66.67)	0.305	12 (6.35)	21 (11.11)	156 (82.54)	1 (11.11)	3 (33.33)	5 (55.56)	0.102
OHIP13	11 (6.36)	25 (14.45)	137 (79.19)	0 (0.00)	2 (7.42)	25 (92.59)	0.213	8 (4.23)	8 (4.23)	173 (91.53)	0 (0.00)	1 (11.11)	8 (88.89)	0.527
OHIP14	7 (4.05)	13 (7.51)	153 (88.44)	0 (0.00)	1 (3.70)	26 (96.30)	0.419	7 (3.70)	11 (5.82)	171 (90.48)	0 (0.00)	1 (11.11)	y (88.89)	0.693

+DMFT and *CPITN: DMF and CPITN were delivered from the broader PhD project. DMF Stands for Decayed, missing teeth due to caries and Filled teeth index. CPITN Stands for Community periodontal index of Treatment needs. They were expressed under yes or no. Yes, indicates participants who experienced caries and periodontal problems respectively and no expressed those who did not experienced dental caries and periodontal problem respectively

*CPITN: CPITN was also provided from the broader PhD project and it Stands for Community periodontal index of Treatment needs. Yes, indicates participants who needed any periodontal treatment and no expressed study participants without any need of periodontal treatment

## Discussion

The results revealed poorer OHRQoL among PLHIV than HIV-negative people in almost all items of OHIP 14**.** Similar results were reported in the literature [18, 19]. HIV infection is known to have a detrimental effect on OHRQoL and this effect may be explained by the HIV infection role in higher prevalence of severe common oral diseases (mostly caries and periodontal disease), as reported by various researchers [20–22]. However, another study done in Uganda, reported no difference in OHRQoL among mothers living with HIV compared to HIV-negative counterparts [23]. This finding may be explained by the fact that pregnant women, regardless of their HIV status, are exposed to oral health problems because of pregnancy changes [24].

They also give less importance to oral health care, mainly because they fear the impact that dental services might have on pregnancy outcomes [18, 25].

Also, in PLHIV and HIV-negative adults, dental caries was significantly associated with poorer OHRQoL in all items of OHIP-14. Consistently with our study, previous researchers revealed the association of dental caries with poorer OHRQoL regardless of HIV status [11, 19, 23, 26–28]. Experiencing dental caries is linked to experiencing various physical, psychological, and social problems, mostly based on the severity of dental caries. For example, untreated caries may lead to aching, inability to eat and rest, and inability to do daily activities or socialize normally [29, 30].

Moreover, dental caries interferes with communication or speaking, social functional, aesthetic, and social damage [28]. In addition, caries may lead to psychological disturbances such as low self-esteem and undermined self-image [28]. In summary, if one can’t eat properly, sleep, perform daily activities, or socialize due to severe dental caries, then general well-being is affected. Therefore, since dental caries touches every single domain of OHIP-14 and leads to poorer OHRQoL, it is essential for the oral health team and all stakeholders to ensure strategies to improve OHRQoL in the Rwandan population, especially among PLHIV.

Similar to our study, previous studies have also reported effect of oral diseases on various OHIP domains among PLHIV compared to general population [12, 26, 31]. For example, a study in Nigeria reported the same domain of physical pain to have the highest impact among PLHIV [12]. Another study in Saudi Arabia reported physical pain as the most affected dimension of OHRQoL [26]. Greater impact on the physical pain and psychological discomfort dimensions may be explained by the impact of DMF experience, which was found to affect significantly PLHIV and HIV-negative adults in this study.

Issues such as aching, lack of comfort to eat, feeling nervous, and being self-consciousness due to oral problems were reported by participants and are linked to dental caries experience among PLHIV and HIV-negative individuals. These results call for a combined effort from policymakers, oral health, and general health teams to ensure oral health programs and strategies to promote oral health and raise awareness on prevention measures to improve population oral health-related quality of life in general and specifically for PLHIV.

Females living with HIV and HIV-negative reported poorer OHRQOL than males”. The literature highlights that males ignore their oral health and rarely visit dentists for prevention services compared to women [32, 33]. These findings suggest that males do not worry much or see dental problems as an important issue for their quality of life.

Surprisingly, none of the 14 oral health impact profile items was significantly associated with periodontal disease assessed through the Community Periodontal Index of Treatment Need (CPITN). Contrary to dental caries, periodontal disease is silent. Regarding periodontal conditions, pain is present, in general, only in cases of severe periodontitis. Since OHRQoL results are the outcome of the self-reported impact of oral conditions on quality of life, this may be why people with painless periodontal disease do not feel concerned about oral issues and do not report the impact on their oral health. As reported for most other non-communicable conditions, it is rare to look for medical help if there is no pain [29]. This issue can lead to late dental visits; in this case, it is when there is advanced periodontal destruction that cannot be reversible even after the treatment. Therefore, oral health education and awareness about periodontal disease and its impact on general health should be key to the general population in Rwanda.

The burden of oral diseases is at high in Rwanda. According to the Rwanda National Oral Health Survey, nearly 65% participants of oral health survey have experienced dental caries with more than 54% untreated caries. In addition, periodontal diseases were found among 60% of participants. Surprisingly, more than 70% of this population have never visited dentists [34]. There should be strategies to engage the Rwandan people to do dental check-ups on time. To ensure healthy dental and periodontium, interventions that tackle oral hygiene practice and engage timely dental visits are needed in Rwanda, especially among high-risk populations such as PLHIV.

The literature reports scarce research regarding OHRQoL of PLHIV and emphasizes the importance of doing so [19, 35]. To the best of our knowledge, there is no single study done in Rwanda on OHRQoL among PLHIV. In addition, there is still a lack of oral health prevention and promotion strategies unique to this high-risk group of PLHIV. This lack of oral health strategies is a major concern to the overall management of PLHIV in Rwanda because oral diseases, mostly caries and periodontal disease, had a significant impact on the general well-being of affected individuals. Oral diseases may negatively affect social interaction, leading to chronic distress, depression, heart disease, and stroke [36]. Moreover, vital functions such as breathing, speaking, selection of what to eat, swallowing, daily work, schooling, and family interaction can all be altered by dental problems [29, 30].

Lacking data regarding OHRQoL in Rwanda is a barrier to adopting strategies to promote oral health and improve the quality of life among Rwandese, especially those living with other chronic diseases, including HIV infection. In addition, the results of this study are essential to inform practice change regarding HIV services by incorporating oral health components to treat PLHIV in Rwanda comprehensively.

### Strength of the study

To our knowledge, this is the first study to look at OHRQoL among PLHIV in Rwanda. In addition, this study considered a comparison group of HIV-negative adults, which is essential to understanding the oral health status of PLHIV in relation to their HIV-negative counterparts, and the comparison component is scarce in previous literature.

## Limitation

This study may have recall bias due to the self-reporting of OHIP data. So, we considered oral impact that occurred within previous 3 months to minimize the recall bias. Also, a cross-sectional study design cannot establish a causal relationship. Moreover, OHRQoL may have suffered influence of HIV-related factors such as HIV infection duration and CD4+ cell counts. We recommend future studies to investigate the influence of HIV-related factors on OHRQoL. Lastly, the study did not look at the association of OHRQoL with other ulcerations or infections or HIV other than caries and periodontal diseases which may affect OHRQoL. We recommend future studies to look at the effect of other ulcerations and infections on OHRQoL.

## Conclusion

Our study revealed poorer OHRQoL among PLHIV than HIV-negative adults. In addition, dental caries was significantly associated with poorer OHRQoL among PLHIV and HIV-negative individuals. Oral health programs are needed to improve the OHRQoL in Rwanda, especially among people at high risk of developing oral disease, like PLHIV. More attention should be given to common oral diseases, such as caries and periodontal diseases. Further similar longitudinal studies and those that consider people with other chronic diseases are needed in Rwanda.

Furthermore, it is essential to consider OHRQoL assessment for further dental research in Rwanda to plan dental public health better. As suggested by other researchers, it is vital to include oral health care as one of the components of the medical health care programs for PLHIV.

## Data Availability

The datasets used and/or analyzed during the current study available from the corresponding author on reasonable request.

## Abbreviations

DMFT: Decayed, Missing due to caries, and Filled Teeth (in the permanent dentition)
CHUK: Kigali Teaching Hospital
CPITN: Community periodontal index of Treatment Needs
HIV: Human immunodeficiency virus
OHIP: Oral Health Impact Profile
OHRQL: Oral Health related Quality of Life
PLHIV: People Living with Human immunodeficiency virus
WHO: World Health Organization
